# Delayed Hyperpigmented Injection Site Reactions Due to Chronic Dupilumab Use

**DOI:** 10.7759/cureus.37441

**Published:** 2023-04-11

**Authors:** Curtis S Pacheco, Kevin M White

**Affiliations:** 1 Department of Internal Medicine, San Antonio Uniformed Services Health Education Consortium, Fort Sam Houston, USA; 2 Department of Allergy and Immunology, Wilford Hall Medical Center, 59th Medical Group, Lackland Air Force Base, USA

**Keywords:** dupilumab hyperpigmented reaction, delayed biologic injection site reaction, atypical biologic injection site reaction, dupilumab dermatologic reaction, delayed dupilumab injection site reaction

## Abstract

Dupilumab is a humanized monoclonal antibody approved for the treatment of chronic rhinosinusitis with nasal polyposis (CRSwNP), asthma, atopic dermatitis, eosinophilic esophagitis, and prurigo nodularis. The most common adverse reactions from dupilumab use are temporary injection site reactions and ocular surface reactions; however, a variety of both acute and delayed cutaneous reactions have also been described. We present a case of delayed hyperpigmented injection site reactions following chronic dupilumab use.

## Introduction

Chronic rhinosinusitis with nasal polyposis (CRSwNP) is a widely prevalent disorder that is estimated to occur in up to 4% of the US population. In patients with aspirin-exacerbated respiratory disease (AERD), nasal polyposis can be particularly severe and difficult to control with both pharmacologic and operative interventions [1]. Dupilumab is a monoclonal antibody that inhibits interleukin 4 (IL-4) receptor alpha and is highly effective in reducing nasal polyp burden, preventing recurrent sinus surgery, and reducing sinus infections in patients with severe CRSwNP, particularly those with AERD [2-4]. The most common adverse reactions from dupilumab use include temporary injection site reactions, ocular surface reactions, and transient eosinophilia [5]. In rare cases, inflammatory arthritis/enthesitis may also occur [6]. Atypical dermatologic reactions such as urticarial reactions, ulcerative injection site reactions, recurrent facial rashes, psoriasis, and alopecia areata have also been described in the medical literature [7-12]. We report a case of delayed hyperpigmented injection site reactions occurring approximately one year after chronic dupilumab use.

## Case presentation

A 28-year-old male with the triad of asthma, non-steroidal anti-inflammatory drug sensitivity, and CRSwNP was evaluated in the Allergy/Immunology clinic for nasal congestion, anosmia, and dysgeusia secondary to recurrent nasal polyposis. The patient was status-post two functional endoscopic sinus surgeries, with the last one approximately six months prior to presentation, which had resulted in only transient improvement of his nasal symptoms despite continued treatment with corticosteroid nasal irrigations and courses of oral corticosteroids postoperatively. Additionally, he had a history of a variety of dermatologic conditions including seborrheic dermatitis, atopic dermatitis, and keloids. He required frequent emollient use and topical corticosteroid courses for the treatment of recurrent eczema. Dupilumab was chosen to treat both his CRSwNP and atopic dermatitis.

**Figure 1:**
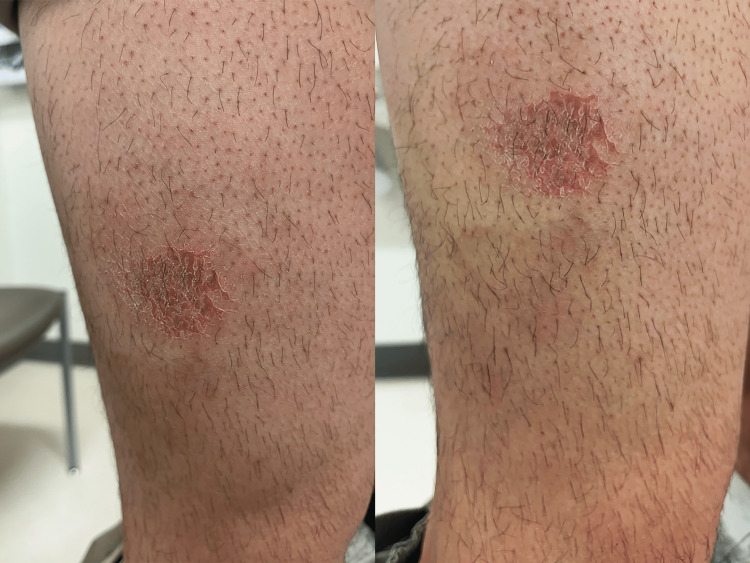
Irregular eczematous lesion on the patient's right thigh two weeks after his most recent subcutaneous dupilumab injection Dupilumab had been injected at the site of the lesion. The figure shows the same lesion from two different viewing angles

The patient was administered dupilumab 600 mg subcutaneously once followed by dupilumab 300 mg subcutaneously every other week for the prevention of recurrent nasal polyp formation and the treatment of atopic dermatitis. After performing subcutaneous injection training and verifying the correct administration technique in the clinic, the patient was allowed to self-administer dupilumab at home with instructions to rotate injection sites between his abdomen and anterior thighs after each administration. After two months of dupilumab use, he reported marked improvement in his upper respiratory symptoms. He had no adverse reactions from dupilumab until approximately one year of continued use, after which he had an abrupt onset of 8-10 cm irregular eczematous patches at dupilumab injection sites. The lesions were asymptomatic, arose almost immediately following injection, and persisted for four to six weeks post-injection before resolving spontaneously (Figure 1).

**Figure 2:**
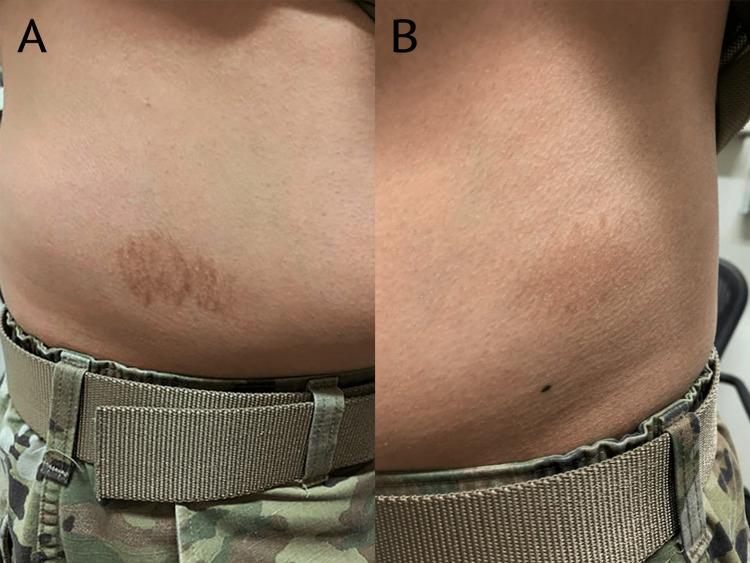
Residual hyperpigmentation at prior dupilumab injection reaction sites approximately six weeks post-injection (A) and eight weeks post-injection (B)

After spontaneous resolution, the lesions left an area of residual hyperpigmentation. Residual hyperpigmented patches continued to persist for up to eight weeks post-injection (Figure 2).

Topical corticosteroids led to no change in the duration of spontaneous resolution. A punch biopsy of a lesion approximately two weeks post-injection was obtained and revealed focal parakeratosis overlying basket weave-orthokeratosis consistent with hyperkeratosis. Additionally, there was focal epidermal spongiosis and dermal perivascular lymphocytic inflammation consistent with minimally spongiotic dermatitis, indicative of atopic dermatitis.

Given the patient was self-administering dupilumab, the proper dupilumab administration technique was verified after the onset of injection site reactions and deemed to be correct. Since the initial onset, his injection site reactions have persisted and continued to occur after each dupilumab administration regardless of the injection site. The reactions have persisted despite transitioning from dupilumab solution to dupilumab autoinjector. The patient elected to continue dupilumab use in spite of these reactions.

## Discussion

We reported a case involving the development of profoundly delayed injection site reactions with chronic dupilumab use. Injection site reactions are common with biologic agents and are among the most common adverse events reported with dupilumab, occurring in approximately 15-18% of patients [13,14]. Typical injection site reactions to biologic agents are characterized by local site swelling, erythema, and pain that often resolve within 48 hours of injection [5]. Our patient had an abrupt onset of asymptomatic eczematous injection site reactions with residual hyperpigmented patches that started approximately one year after the initiation of dupilumab. A myriad of idiosyncratic injection site reactions associated with dupilumab has been reported, including delayed urticarial reactions occurring months after initiating treatment [7,8,15]. To our knowledge, this is the first report of injection site reaction to dupilumab occurring approximately one year into treatment.

It is unclear as to why our patient developed this reaction so far into his treatment course after otherwise tolerating dupilumab without difficulties. There had been no changes to his dupilumab dose, dosing schedule, clinical status, or additional medication regimen that would suggest an alternative explanation for his injection site reactions. As stated previously, his reactions have persisted despite the use of both dupilumab autoinjector and dupilumab solution. Skin biopsy findings of an active lesion were indicative of atopic dermatitis but did not reveal findings consistent with a specific hypersensitivity reaction. His residual hyperpigmented patches at prior injection reaction sites are similar to those of post-inflammatory hyperpigmentation, which is characterized by patchy areas of hyperpigmentation caused by the stimulation of melanocytes by inflammatory mediators due to either an intrinsic or extrinsic trigger [16]. Given that dupilumab inhibits IL-4, IL-13, and, in turn, type 2 (Th2) inflammation, it is possible that local changes in inflammatory pathways at injection sites after prolonged dupilumab use may have resulted in abnormal cutaneous inflammation after subsequent injections, thereby causing his clinical presentation [17]. Polysorbates are frequently used as stabilizing agents in biologic agents, which has led to the implication of polysorbate degradation products as the etiology of injection site reactions to biologic agents [7]. We do not suspect polysorbates or their degradation products played a role in the etiology of our patient's injection site reactions given that he had received polysorbates in various forms in vaccinations, which had not resulted in an injection site reaction. Additionally, his immensely delayed injection site reaction precludes polysorbates as the etiology. We hypothesize that his underlying atopy and propensity for forming keloids may have contributed to the development of this reaction, given that dysregulated inflammatory pathways are also implicated in the pathophysiology of keloids/hypertrophic scars [18].

## Conclusions

A wide array of injection site reactions has been observed with the use of subcutaneous biologic agents. Reactions may be acute, sub-acute, or even delayed by several months, as observed in our case. The decision to continue therapy in spite of an injection site reaction is individualized and depends on the severity of the reaction, patient preference, and provider assessment. This case report serves to further highlight the atypical injection site reactions that may be seen with dupilumab use.
